# m^6^A reader IGF2BP1 accelerates apoptosis of high glucose-induced vascular endothelial cells in a m^6^A-HMGB1 dependent manner

**DOI:** 10.7717/peerj.14954

**Published:** 2023-03-27

**Authors:** Anru Liang, Jianyu Liu, Yanlin Wei, Yuan Liao, Fangxiao Wu, Jiang Ruan, Junjun Li

**Affiliations:** 1 Department of Burns and Plastic Surgery, The Third Affiliated Hospital of Guangxi Medical University and The Second People’s Hospital of Nanning, Nanning, China; 2 Department of Clinical Laboratory, Guiping People’s Hospital, Guigping, China; 3 Department of Emergency, The People’s Hospital of Guangxi Zhuang Autonomous Region & Guangxi Academy of Medical Sciences, Nanning, China; 4 Research Center of Medical Sciences, The People’s Hospital of Guangxi Zhuang Autonomous Region & Guangxi Academy of Medical Sciences, Nanning, China

**Keywords:** N^6^-methyladenosine, IGF2BP1, Vascular endothelial cells

## Abstract

Emerging evidence indicates that N^6^-methyladenosine (m^6^A) plays a critical role in vascular biological characteristic. In diabetes mellitus pathophysiology, high glucose (HG)-induced vascular endothelial dysfunction is associated with diabetes vascular complications. Nevertheless, the underlying mechanism of high glucose (HG)-related m^6^A regulation on vascular endothelial cells is still unclear. Results indicated that m^6^A reader insulin-like growth factor 2 mRNA-binding protein 1 (IGF2BP1) was up-regulated in HG-treated human umbilical vascular endothelium cells (HUVECs) comparing to normal group. Functionally, results indicated that IGF2BP1 knockdown recovered the proliferation of HUVECs inhibited by HG-administration. Besides, IGF2BP1 knockdown reduced the apoptosis induced by HG-administration. Mechanistically, IGF2BP1 interacted with HMGB1 mRNA and stabilized its expression of m^6^A-modified RNA. Therefore, these findings provided compelling evidence demonstrating that m^6^A reader IGF2BP1 contributes to the proliferation and apoptosis of vascular endothelial cells in hyperglycaemia, serving as a target for development of diabetic angiopathy therapeutics.

## Introduction

Diabetes mellitus (DM) is a multifactorial metabolic trait and chronic pathophysiological process (Cheok et al., 2020; Hayashi, Rakugi & Morishita, 2020). Multiple stimuli, including high glucose (HG), could result in endothelial dysfunctions (Vergès, 2020). In vascular homeostasis, vascular endothelium consists of endothelial cells and acts as the main barrier to maintain vascular permeability (Beazer et al., 2020; Cheng & Kishore, 2020). HG-induced vascular endothelial dysfunction contributes to multiple vascular metabolic disorders, including coronary artery disease, atherosclerosis, diabetic nephropathy and others (Wautier & Wautier, 2020).

N^6^-methyladenosine (m^6^A) acts as the most prevalent type of methylations occurred on RNA, which has become a hotspot in the epigenetic research community (Lu et al., 2021; Su et al., 2021). The m^6^A is a methylation at N^6^ position of adenosine and enriched in this RRACH consensus sequence (R: A or G; A: m6A; and H: A, C, U) (Xu et al., 2021; Zhou et al., 2021). The biological functions of m^6^A modification were regulated by three core proteins: writers (methyltransferases), erasers (demethylases) and readers (m^6^A binding proteins). For the HG-induced vascular endothelium dysfunction, m^6^A plays critical roles. For instance, in oxidized low-density lipoprotein (ox-LDL)--induced human umbilical vascular endothelium cells (HUVECs), methyltransferase-like 3 (METTL3) knockdown inhibits the cellular tube formation, proliferation, migration and VEGF secretion and prevents *in vivo* embryos angiogenesis (Dong et al., 2021). Moreover, METTL14/METTL3 upregulates in ox-LDL treated HUVECs and the knockdown of METTL14/METTL3 increases bcl-2 expression level and viability of ox-LDL-incubated cells (Liu et al., 2022). Thus, these data suggest the essential role of m^6^A in vascular endothelium dysfunction.

Here, our research aimed to address these questions by determining m^6^A-associated reader insulin-like growth factor 2 mRNA-binding protein 1 (IGF2BP1) expression patterns in HG-induced HUVECs. Consequently, IGF2BP1 emerged as highly expressed m^6^A reader in HG-induced HUVECs and the IGF2BP1 dysregulated expression dramatically modulated the proliferation and apoptosis. Interestingly, IGF2BP1 regulated the progression of HG-induced HUVECs by changing the stability of HMGB1 mRNA in an m^6^A-dependent manner.

## Materials and Methods

### Cell culture and diabetes model treatment

Human umbilical vascular endothelium cells (HUVEC) were purchased from ScienCell Research Laboratories (Carlsbad, CA, USA) and maintained in Endothelial Cell Medium (ECM, Carlsbad, CA, USA) added with 10% fetal bovine serum (FBS; Gibco, Billings, MT, USA) and 5.6 mmol/L glucose. The diabetes model treatment was performed as previously described (Li et al., 2015; Zhao et al., 2022). For diabetes group (high glucose, HG), HUVECs were exposed to 30 mmol/L d-glucose. For control group (normal glucose, NG), HUVECs were cultured in culture medium with 5.6 mmol/L d-glucose and 24.4 mmol/L mannitol.

### Plasmids construction and cell transfections

To construct silenced expression plasmids of IGF2BP1, the sequences of shRNA targeting IGF2BP1 and corresponding controls (sh-NC) were amplified respectively by purchased from GeneChem (Shanghai, China), and then cloned into the HUVECs following the manufacturer-recommended protocol.

### RNA extraction and qRT‑PCR

Total RNA in cells was extracted in accordance with the manual provided with the TRIzol reagent (Thermo Fisher, Waltham, MA, USA). The extracted total RNA was treated with RNase-free DNase and its reverse transcription was performed in accordance with the manual provided ReverTra Ace qPCR RT Master Mix with gDNA Remover (Toyobo, Osaka, Japan). qPCR was performed in accordance with the manual provided by SYBR Green PCR kit (TaKaRa, Dalian, China) on Applied Biosystems 7300. After the reactions, the cycle threshold (CT) data were determined using fixed thresholds setting, and the mean CT value was determined from triplicate PCR. The primers were listed in Table S1.

### Western blot assay

Total protein in HUVECs was extracted using RIPA buffer containing sodium chloride (NaCl, 150 mM), Tris-hydrochloride (HCl, 50 mM), 1 mM sodium fluoride, ethylenediaminetetraacetic acid (EDTA, 5 mM), 1% Triton X-100, 1% deoxycholate, 1 mM sodium vanadate and a protease inhibitor cocktail. The quality was quantified using BCA method (Thermo Fisher, Waltham, MA, USA). Subsequently, the protein was electrophoresed on sodium dodecyl sulfate-polyacrylamide gel electrophoresis (SDS-PAGE) gels and transferred to polyvinylidene fluoride (PVDF) membrane (Millipore, Burlington, MA, USA). PVDF membranes were blocked with 5% BSA for 1 h at room temperature and then incubated at 4 °C overnight with anti-IGF2BP1 (cat. D33A2, #8482, dilution of 1:1,000; Cell Signaling Technology, Danvers, MA, USA), anti-HMGB1 (cat. D3E5, #6893, dilution of 1:1,000; Cell Signaling Technology, Danvers, MA, USA), anti-β-Actin (cat. 8H10D10, #3700, dilution of 1:1,000; Cell Signaling Technology, Danvers, MA, USA). After incubation with the horseradish peroxidase-labeled goat anti-rabbit IgG secondary antibody (ab6721, cat. 1:2,000; Abcam, Cambridge, MA, USA), the PVDF membranes were visualized with enhanced chemiluminescence system kit (Millipore, Burlington, MA, USA) according to the manufacturer’s protocol.

### Cell counting kit‑8 (CCK8) assay

Cell proliferation was determined by CCK-8 (Beyotime, Shanghai, China). HUVECs were differently treated (HG or NG or transfection) were cultured in a 96-well plate for 0, 24, 48, 72 h respectively and then incubated with CCK-8 kit. The proliferation was determined *via* the absorbance at 450 nm by microplate reader (Thermo Fisher Scientific, Waltham, MA, USA).

### Cellular apoptosis analysis

The HUVEC cells were harvested and collected after indicated treatment. The, cells then were resuspended with pre-cold PBS (50 µl). After 30 min, the apoptotic cells were calculated by Annexin V-FITC Apoptosis Detection Kit (Beyotime, Shanghai, China) with flow cytometry.

### Ethynyl-2-deoxyuridine (EdU) incorporation assay

EdU assay was performed to determine the proliferation of HUVECs. In brief, transfected HUVECs and corresponding RNA were incubated with EdU (100 μl of 50 μM) (Ribo Bio, Guangzhou, China) according to the Ribo Bio’s instructions per well at 37 °C for 2 h, respectively. The EdU incorporation rate was calculated as EdU-positive cells ratio to total Hoechst-positive cells (blue cells). The cells were counted using Image-Pro Plus (IPP) 6.0 software (Media Cybernetics, Rockville, MD, USA).

### m^6^A quantification assay

The global m6A levels in mRNA were measured by EpiQuik m^6^A RNA Methylation Quantification Kit (Colorimetrically; Epigentek, Farmingdale, NY, USA) following the manufacturer’s protocol. Poly-A-purified RNA (200 ng) was used for each sample analysis. The m^6^A levels were colorimetrically quantified by reading each well absorbance at 450 nm wavelength, and calculated based on the standard curve.

### RNA immunoprecipitation

RIP was performed to determine the interaction within IGF2BP1 and HMGB1 mRNA. HUVECs stably silencing IGF2BP1 and (sh-IGF2BP1) control cells (sh-NC) were lysed with radioimmunoprecipitation (RIP) lysis buffer (Magna RIP Kit; Millipore, Burlington, MA, USA) at 4 °C *via* disruptive sonication. Endogenous HMGB1 mRNA immunoprecipitations were performed using an anti-IGF2BP1 antibody (Abcam, Cambridge, MA, USA) overnight at 4 °C. The immunoprecipitated protein-RNA complex was subjected to quantitative real-time polymerase chain reaction (qRT-PCR) using primers and normalizing to input.

### m^6^A methylated RNA immunoprecipitation-PCR

MeRIP-PCR was performed for the quantification of m^6^A-modified HMGB1 mRNA. Total RNA was isolated from HUVECs by Trizol, and anti-m^6^A antibody (cat. ABE572, 3 μg; Millipore, Burlington, MA, USA) or anti-IgG (Cell Signaling Technology, Danvers, MA, USA) was conjugated to protein A/G magnetic beads in IP buffer (140 mM NaCl, 20 mM Tris pH 7.5, 2 mM EDTA, 1% NP-40). Total RNA (100 μg) was incubated with antibody in IP buffer supplemented with RNase inhibitor and protease inhibitor. After of the incubation, beads were eluted for further qRT-PCR assay.

### RNA stability

The HMGB1 mRNA stability was detected by Actinomycin D administration. In brief, the actinomycin D (Act-D; Sigma-Aldrich, St. Louis, MO, USA, 5 μg/ml) was added to HUVEC cells. After harvesting of HUVECs, the RNA was isolated by TRIzol for qRT-PCR analysis. The half-life of HMGB1 mRNA was calculated normalizing to GAPDH data.

### Statistical analysis

All the analysis was performed using GraphPad Prism V8.0 and SPSS V22.0. Data was calculated and displayed as Mean ± standard deviation (SD). The t-test and $\chi^2$-test were used to analyze the differences between different groups. *P*-value less than 0.05 was considered as statistical significance (**p < 0.01,*p < 0.05). All *in vitro* experiments were performed in triplicate and were repeated three times.

## Results

### IGF2BP1 was up-regulated in HG-induced HUVECs

**Figure 1:**
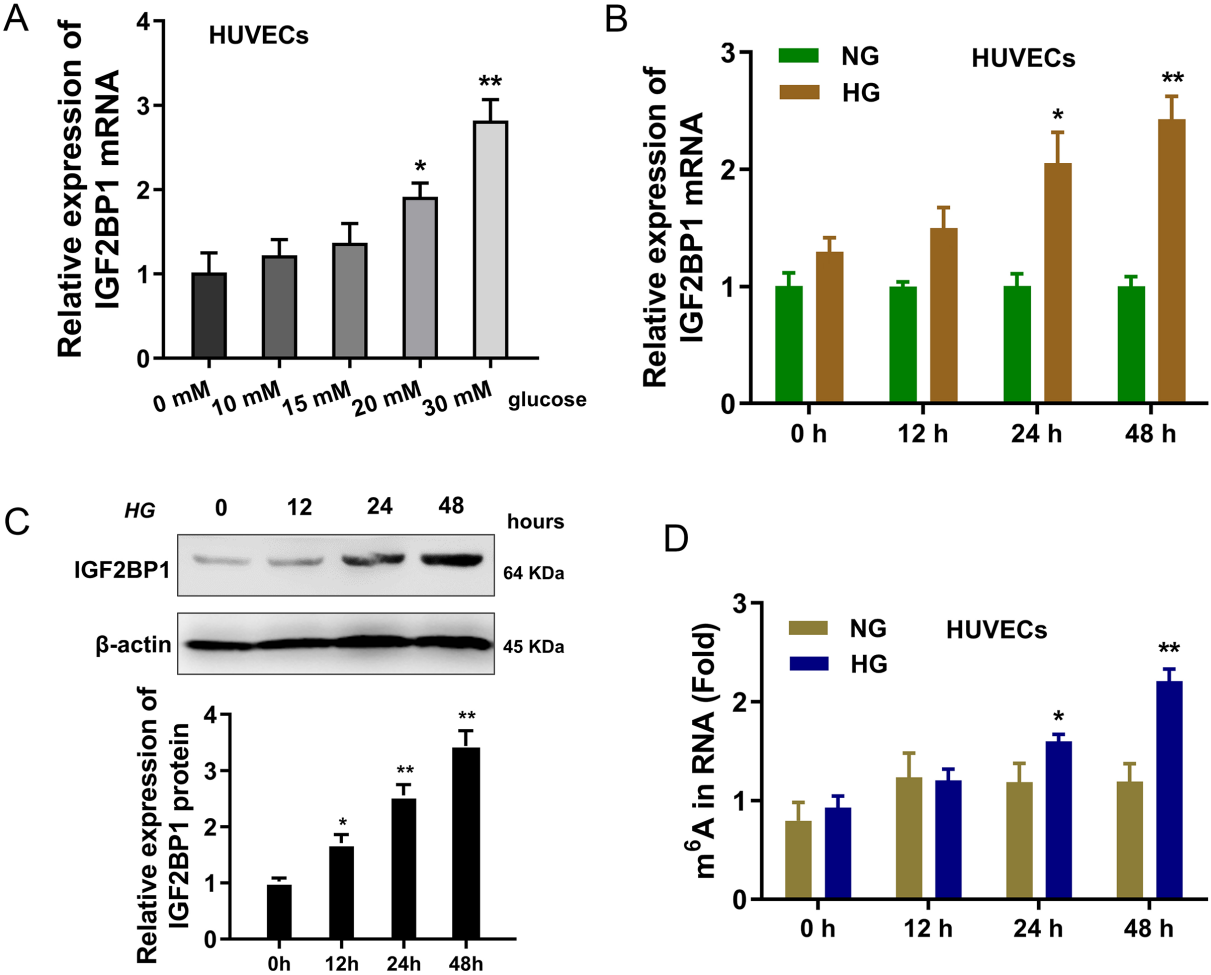
IGF2BP1 was up-regulated in the HG-induced HUVECs. (A) RT-qPCR assay was performed to detect the IGF2BP1 mRNA in HUVECs treated by HG administration (0, 10, 15, 20, 30 mM). (B) RT-qPCR assay was performed to detect the IGF2BP1 mRNA in HUVECs treated by HG administration (0, 12, 24, 48 h). (C) Western blotting assay was performed to determine the IGF2BP1 protein level in HUVECs treated by HG administration (0, 12, 24, 48 h). (D) The m^6^A modification analysis detected the m^6^A level in HUVECs treated by HG administration (0, 12, 24, 48 h). *p < 0.05, **p < 0.01. All *in vitro* experiments were performed in triplicate and were repeated three times.

In present research, the cellular diabetes mellitus model was constructed in human umbilical vascular endothelium cells (HUVEC) with HG administration. Results indicated that the expression of IGF2BP1 mRNA was up-regulated in HUVECs with increasing dosage HG treatment (0, 10, 15, 20, 30 mM) (Fig. 1A). Then, with HG administration (30 mM), the expression of IGF2BP1 mRNA was up-regulated with time increasing (0, 12, 24, 48 h) (Fig. 1B). Moreover, the expression of IGF2BP1 protein was up-regulated as treatment time increasing (0, 12, 24, 48 h) (Fig. 1C). Furthermore, in HG-treated HUVECs, the m^6^A modification was significantly up-regulated (Fig. 1D). Overall, these findings suggested that IGF2BP1 expression was up-regulated in the HG-treated HUVECs.

### Knockdown of IGF2BP1 mitigated HG-induced apoptosis of HUVECs

**Figure 2:**
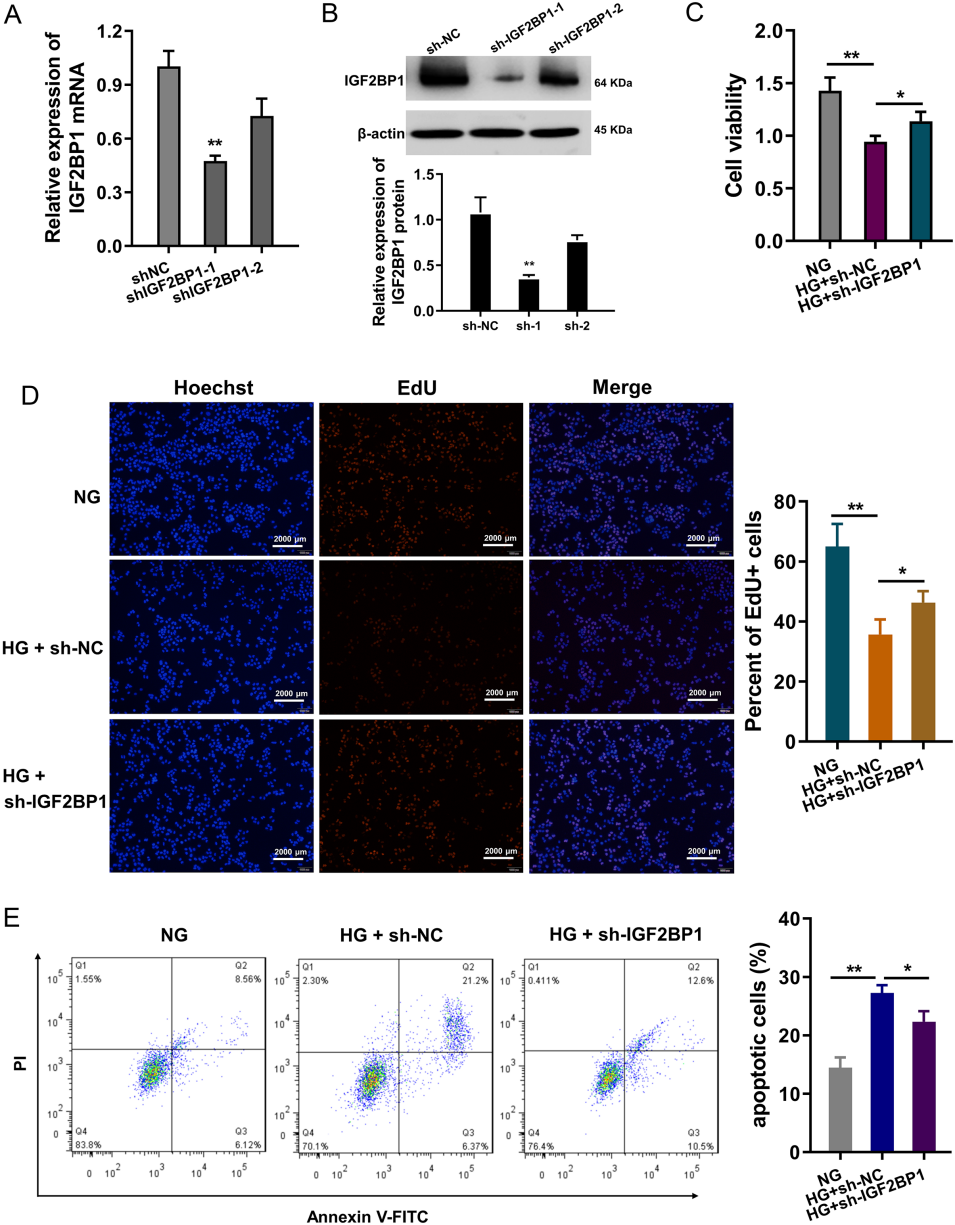
Knockdown of IGF2BP1 mitigated the HG-induced apoptosis of HUVECs. (A) RT-PCR and (B) western blotting analysis were respectively performed to detect the IGF2BP1 mRNA or protein levels in HG-induced HUVECs. (C) Cellular viability analysis by CCK-8 assays was performed for HUVECs’ viability. (D) EdU assay showed the cellular proliferation of HUVECs with HG administration upon IGF2BP1 knockdown or control. (E) Apoptosis analysis by flow cytometry revealed the apoptosis of HUVECs transfected with IGF2BP1 knockdown or control. *p < 0.05; **p < 0.01. All *in vitro* experiments were performed in triplicate and were repeated three times.

In HG-treated HUVECs, functional assays were performed to investigate the roles of IGF2BP1. The knockdown of IGF2BP1 was performed in HUVECs, and the efficient was examined by RT-PCR (Fig. 2A) and western blot (Fig. 2B). Cellular viability analysis found that HG administration reduced the cellular viability, and IGF2BP1 knockdown recovered the viability (Fig. 2C). For the proliferation of HUVECs, EdU assay indicated that HG administration reduced the cellular proliferation, and the IGF2BP1 knockdown facilitated the proliferation (Fig. 2D). Apoptosis analysis found that HG administration promoted the apoptosis of HUVECs, and IGF2BP1 knockdown reduced the apoptosis (Fig. 2E). Overall, these date suggested that knockdown of IGF2BP1 mitigated HG-induced apoptosis of HUVECs.

### HMGB1 acted as the target of IGF2BP1 by m^6^A modified sites on HMGB1 mRNA 

**Figure 3:**
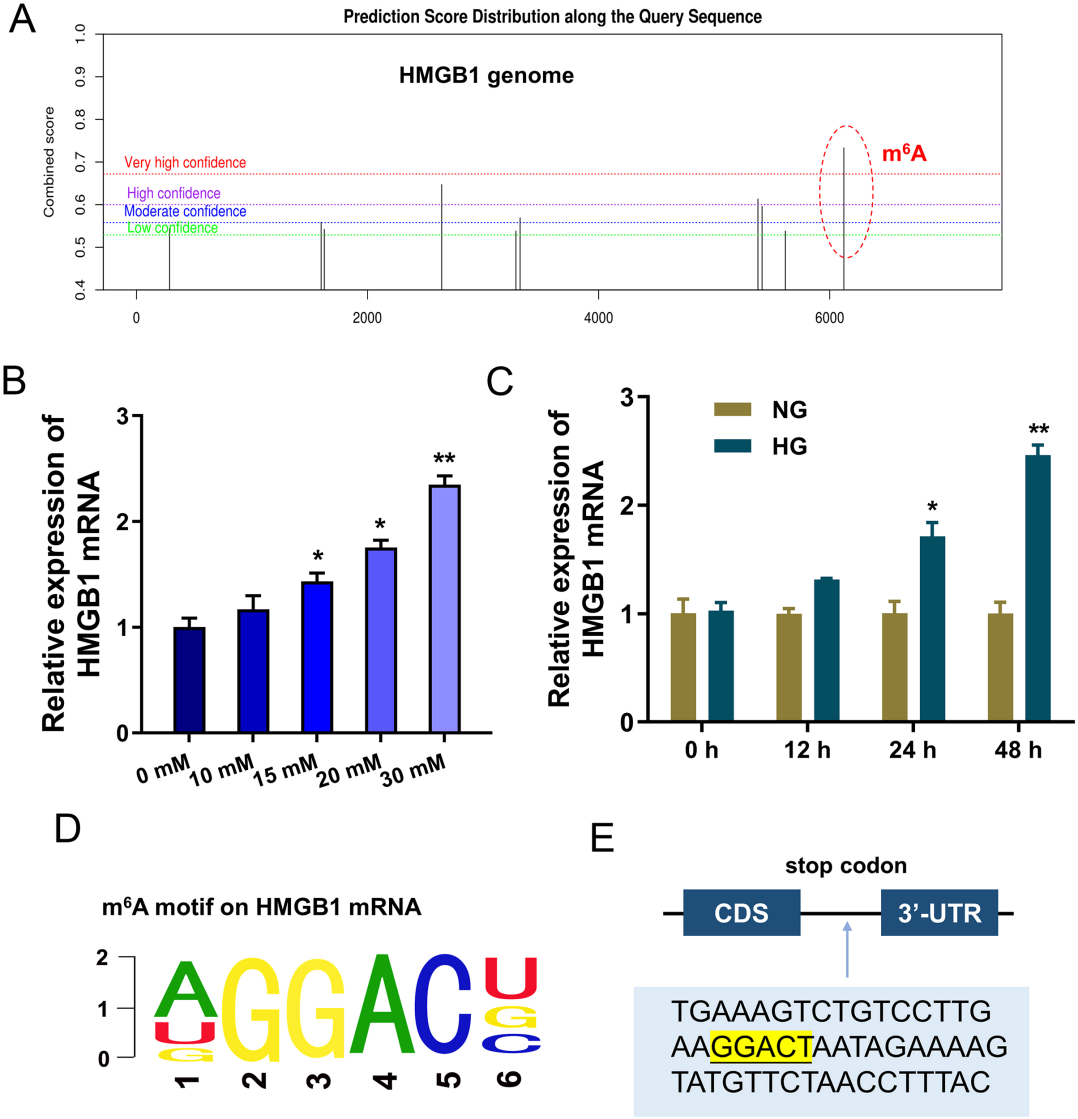
HMGB1 acted as the target of IGF2BP1 by m^6^A modified sites on HMGB1 mRNA. (A) Bioinformatics prediction online system (SRAMP, http://www.cuilab.cn/sramp) was performed to analyze the binding targets of IGF2BP1. (B) RT-PCR analysis revealed the expression of HMGB1 mRNA in HUVECs with HG increasing dosage (0, 10, 15, 20, 30 mM). (C) RT-PCR analysis revealed the expression of HMGB1 mRNA in HUVECs with HG administration (30 mM) as the treatment time increasing (0, 12, 24, 48 h). (D) The m^6^A motif of IGF2BP1 on HMGB1 genome was GGAC. (E) BLAST analysis was performed to reveal the m^6^A modified site in the 3′-UTR of HMGB1 mRNA. *p < 0.05; **p < 0.01. All *in vitro* experiments were performed in triplicate and were repeated three times.

To discover the potential downstream target of IGF2BP1, we took advantage of bioinformatics prediction online system (SRAMP, http://www.cuilab.cn/sramp) to analyze its binding targets. Results inspired that there was a significant m^6^A site on HMGB1 genome (Fig. 3A). Results indicated that HMGB1 mRNA expression was up-regulated with HG dosage increasing (0, 10, 15, 20, 30 mM) (Fig. 3B). Furthermore, with HG treatment (30 mM), HMGB1 mRNA expression was up-regulated along treatment time increasing (0, 12, 24, 48 h) (Fig. 3C). The m^6^A motif of IGF2BP1 on HMGB1 genome was GGAC (Fig. 3D). In the 3′-UTR of HMGB1 mRNA, blast analysis revealed the m^6^A modified site (Fig. 3E). Collectively, these data suggested that HMGB1 acted as the target of IGF2BP1 by m^6^A modified sites on HMGB1 mRNA.

### IGF2BP1 enhanced the stability of HMGB1 mRNA *via* m^6^A-dependent manner

**Figure 4:**
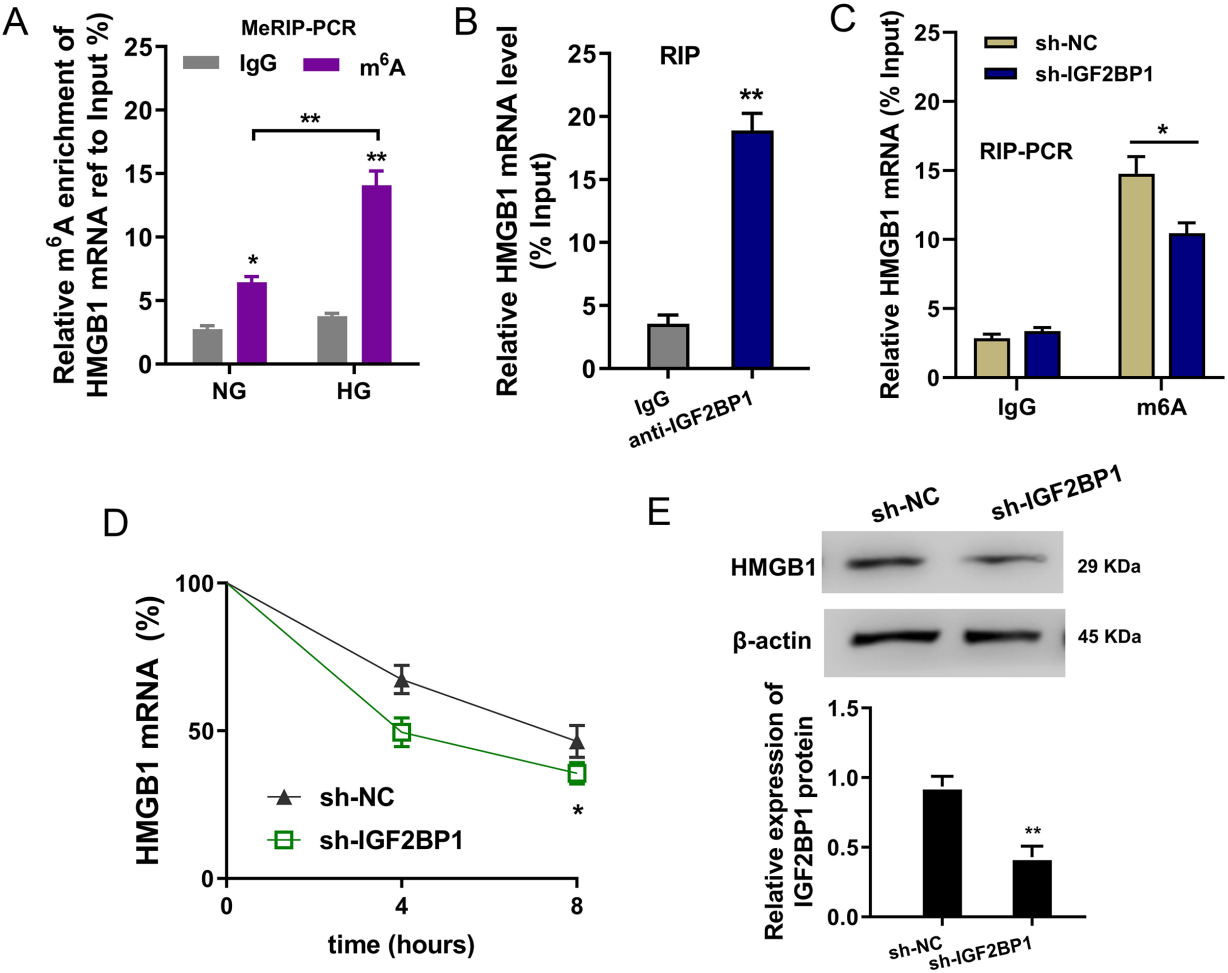
IGF2BP1 enhanced the stability of HMGB1 mRNA *via* m^6^A-dependent manner. (A) MeRIP-PCR was performed to detect the m^6^A modified enrichment on HMGB1 mRNA using anti-m^6^A antibody in the HG administration HUVECs. (B) RIP-PCR assay was performed to detect the interaction within HMGB1 mRNA and IGF2BP1 using anti-IGF2BP1 antibody. (C) RIP-PCR assay was performed to detect the interaction within HMGB1 mRNA and IGF2BP1 in HUVECs transfected with IGF2BP1 shRNA (sh-IGF2BP1) and control (sh-NC). (D) RNA stability assay following qPCR was performed to detect the HMGB1 mRNA remaining in Act D administration in HUVECs transfected with IGF2BP1 shRNA (sh-IGF2BP1) and control (sh-NC). (E) Western blot assay was performed to identify the HMGB1 protein in HUVECs transfected with IGF2BP1 shRNA (sh-IGF2BP1) and control (sh-NC). *p < 0.05; **p < 0.01. All *in vitro* experiments were performed in triplicate and were repeated three times.

In the HG administration HUVECs, MeRIP-PCR was performed to detect the m^6^A modified enrichment on HMGB1 mRNA. Results suggested that the m^6^A enrichment of HMGB1 mRNA was elevated upon HG administration (Fig. 4A). Moreover, the interaction within HMGB1 mRNA and IGF2BP1 was identified using RIP-PCR, and results indicated that HMGB1 mRNA remarkably bound with IGF2BP1 in HUVECs (Fig. 4B). Furthermore, the precipitated HMGB1 mRNA enrichment was reduced in the IGF2BP1 knockdown (Fig. 4C). RNA stability assay indicated that IGF2BP1 knockdown decreased the HMGB1 mRNA remaining in Act D administration in HUVECs (Fig. 4D). Then, the HMGB1 protein level was decreased in IGF2BP1 knockdown in HUVECs (Fig. 4E). Collectively, these data suggested that IGF2BP1 enhanced the stability of HMGB1 mRNA *via* m^6^A-dependent manner.

## Discussion

Endothelial cells apoptosis is one of the main biochemical characteristics of endothelial dysfunction, which is triggered by various stimulations, including high glucose, hypoxia, oxidized low density lipoproteins, oxidative stress and angiotensin II (Lin et al., 2022; Luchetti et al., 2017). In vascular endothelial cells, high glucose could accelerate the apoptosis and aggravate the abnormity (Barnes, Mesarwi & Sanchez-Azofra, 2022; Caporali et al., 2022).

N^6^-methyladenosine (m^6^A), the most common RNA chemical modification on posttranscription, could participate in numerous pathophysiological processes (Dhawan et al., 2022; Ye et al., 2022). In the vasculopathy, more and more literatures have indicated the essential roles of m^6^A. For instance, m^6^A methyltransferase METTL14 plays major roles in TNF-α-induced endothelial cell inflammation through directly targeting m^6^A modification of important transcription factor FOXO1. METTL14 enhances FIXO1 translation through subsequent YTHDF1 recognition (Jian et al., 2020). Regarding to m^6^A methyltransferase METTL3, the silencing or overexpression of METTL3 altered the endothelial cell viability/proliferation/migration/tube formation through regulating Wnt signaling *via* the m^6^A modification of target genes (LRP6, DVL1) to enhance the translation of LRP6 and DVL1 in an YTHDF1-dependent manner (Yao et al., 2020). Collectively, these studies suggest that m^6^A-mediated modification play an important mechanism in HG-related Vascular pathology.

Here, our work focused on the functions of m^6^A reader IGF2BP1 on the blood vessel endothelium. We found that IGF2BP1 levels increased upon HG administration. The knockdown of IGF2BP1mitigated the HG-induced apoptosis of HUVECs, besides, IGF2BP1 knockdown renewed the proliferation. Thus, based on our results, we concluded that IGF2BP1 could remarkably regulate the HG-induced vascular pathophysiology.

Given that IGF2BP1 regulated the apoptosis and proliferation of HUVECs, we utilized this discovery to further explore the undergoing mechanism. Interestingly, we found that IGF2BP1 directly bound with the HMGB1 mRNA *via* m^6^A modification site. Moreover, IGF2BP1 enhanced the stability of HMGB1 mRNA to up-regulate its protein outcome. In the endothelial cell injury, HMGB1 has been reported to regulate the apoptosis (Zhang & Liu, 2021), inflammation (Foglio et al., 2022) and autophagy (Feng et al., 2022) of vascular endothelial cell. Thus, these data suggested the critical roles of HMGB1 in pathological changes of blood vessels.

As the mechanism of the relationship between inflammatory response and atherosclerosis, m^6^A has become a novel focus in the clinical therapeutic strategy for diabetes mellitus. m^6^A-dependent post-transcription modification may be a target for diabetes mellitus therapy. Here, we utilized the bio-functional assays to investigate that whether IGF2BP1 and m^6^A can affect the phenotypic modulation of HUVECs through m^6^A modification. IGF2BP1 regulates the high glucose-induced vascular endothelial cells apoptosis *via* m^6^A/HMGB1 axis by m^6^A-dependent manner. For the limitation of this cell line, this study utilized HUVECs with HG-treatment to model diabetic endothelial dysfunctions. Limited by the experiment condition and epidemic, there are a lot of defects and insufficient for the assay data and design. Such as it is, this finding still provides a instructive insight for m^6^A and diabetic endothelial dysfunctions.

**Figure 5:**
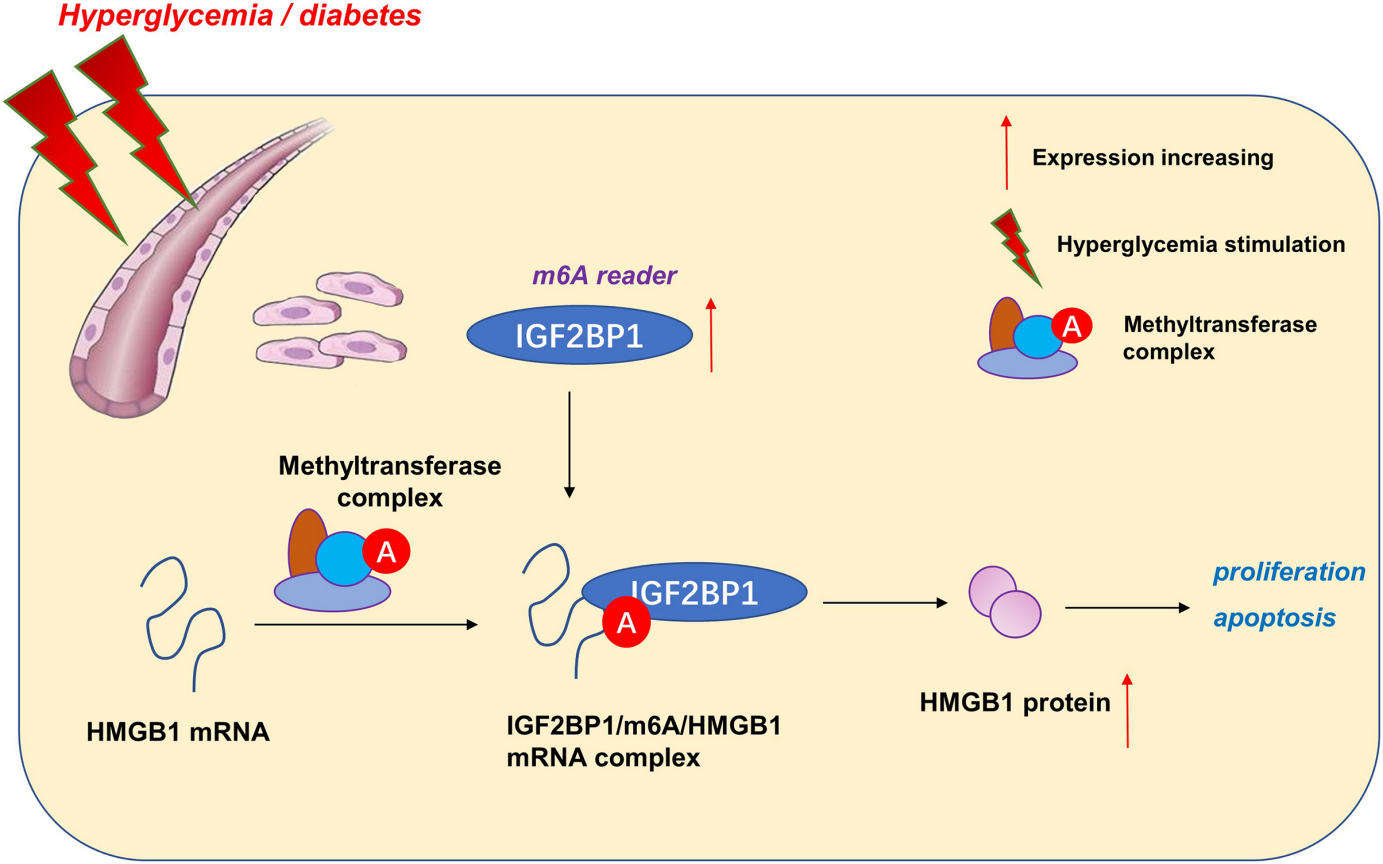
IGF2BP1/m^6^A/HMGB1 axis regulates high glucose-induced vascular endothelial cells apoptosis *via* m^6^A-dependent manner.

Taken together, our data provide robust evidence that IGF2BP1 is an efficient regulator in HG-induced HUVECs. IGF2BP1 and m^6^A-dependent modification may be one of the primary pathogenesis of vascular pathology and hyperglycemia (Fig. 5). These findings strongly support an integral role for m6A in vessel homeostasis and accelerate high glucose-induced dysfunction of endothelial cells.

## Supplemental Information

10.7717/peerj.14954/supp-1 Supplemental Information 1 Primer sequences for qRT-PCR and sequences of shRNA.

10.7717/peerj.14954/supp-2 Supplemental Information 2 Label for WB blot.

10.7717/peerj.14954/supp-3 Supplemental Information 3 Uncropped blots.

10.7717/peerj.14954/supp-4 Supplemental Information 4 PCR Data.

## Additional Information and Declarations

### Data Availability

The following information was supplied regarding data availability: The raw data are available as Supplemental Files.
