# Supplementary material for: m6A reader IGF2BP1 accelerates apoptosis of high glucose-induced vascular endothelial cells in a m6A-HMGB1 dependent manner
Source: PeerJ. 2023 Mar 27;11:e14954. doi: 10.7717/peerj.14954 (PMC10062336; doi:10.7717/peerj.14954)
Supplement: Supplemental Information 1 [file peerj-11-14954-s001.docx]

**Table S1**. Primers sequences for qRT-PCR and sequences of shRNA.

|  | Sequences |
| --- | --- |
| IGF2BP1 | forward, 5’-CAAAGGAGCCGGAAAATTCAAAT-3’  reverse, 5’-CGTCTCACTCTCGGTGTTCA-3’ |
| HMGB1 | forward, 5’-TATGGCAAAAGCGGACAAGG-3’  reverse, 5’-CTTCGCAACATCACCAATGGA -3’ |
| sh-IGF2BP1-1 | 5’- CGTGTTCAGATCTGGAAAATT-3’ |
| sh-IGF2BP1-2 | 5’-GCGAGGGGTAAATGTGACTTT-3’ |
| GAPDH | forward, 5’-CTGGGCTACACTGAGCACC -3’  reverse, 5’-AAGTGGTCGTTGAGGGCAATG-3’ |
