# Supplementary figures and images for: m6A reader IGF2BP1 accelerates apoptosis of high glucose-induced vascular endothelial cells in a m6A-HMGB1 dependent manner
Source: PeerJ. 2023 Mar 27;11:e14954. doi: 10.7717/peerj.14954 (PMC10062336; doi:10.7717/peerj.14954)

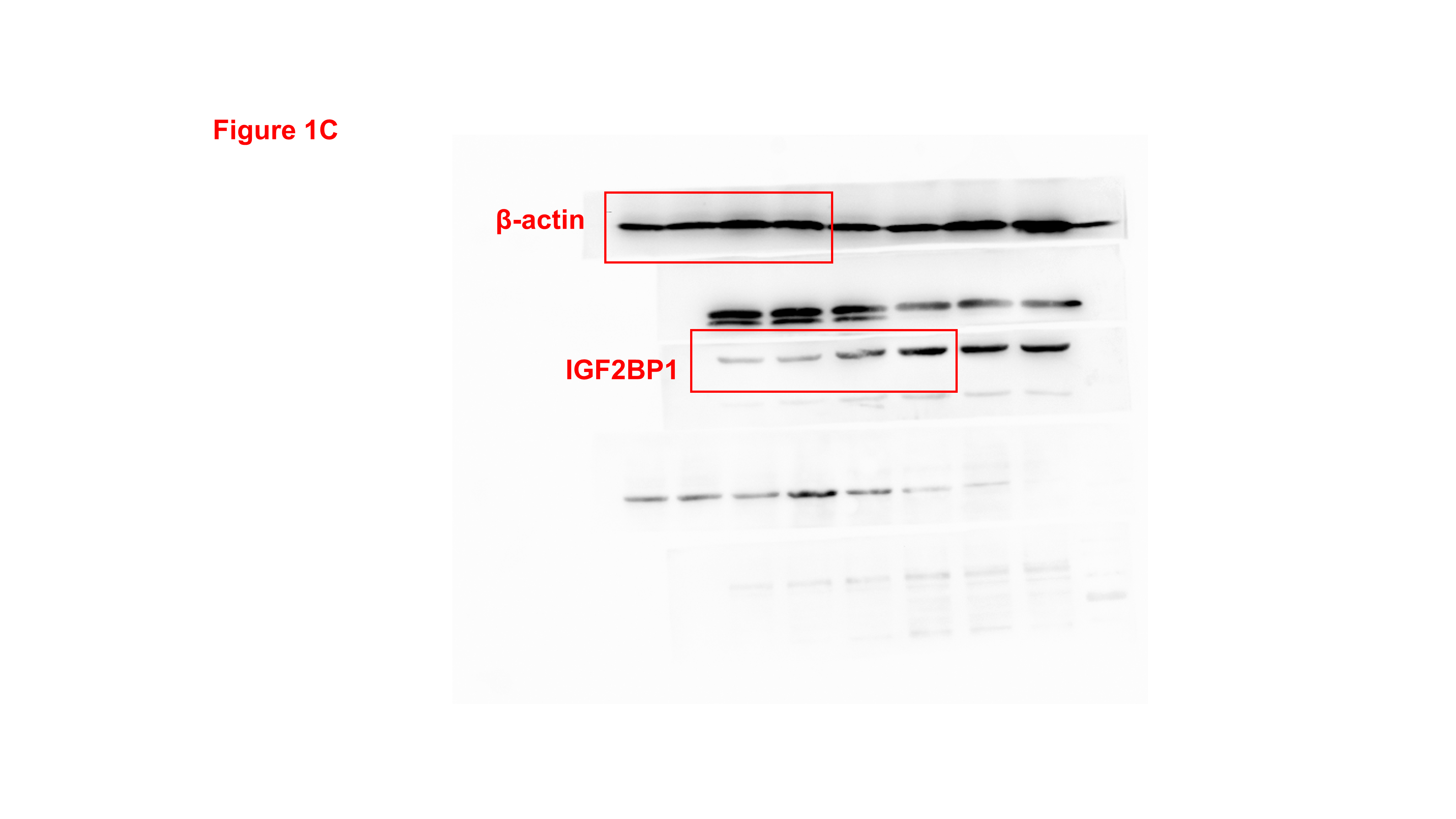

Supplement: Supplemental Information 2 [file peerj-11-14954-s002.zip › Label for WB blot/WB in Fig 1C .jpg]

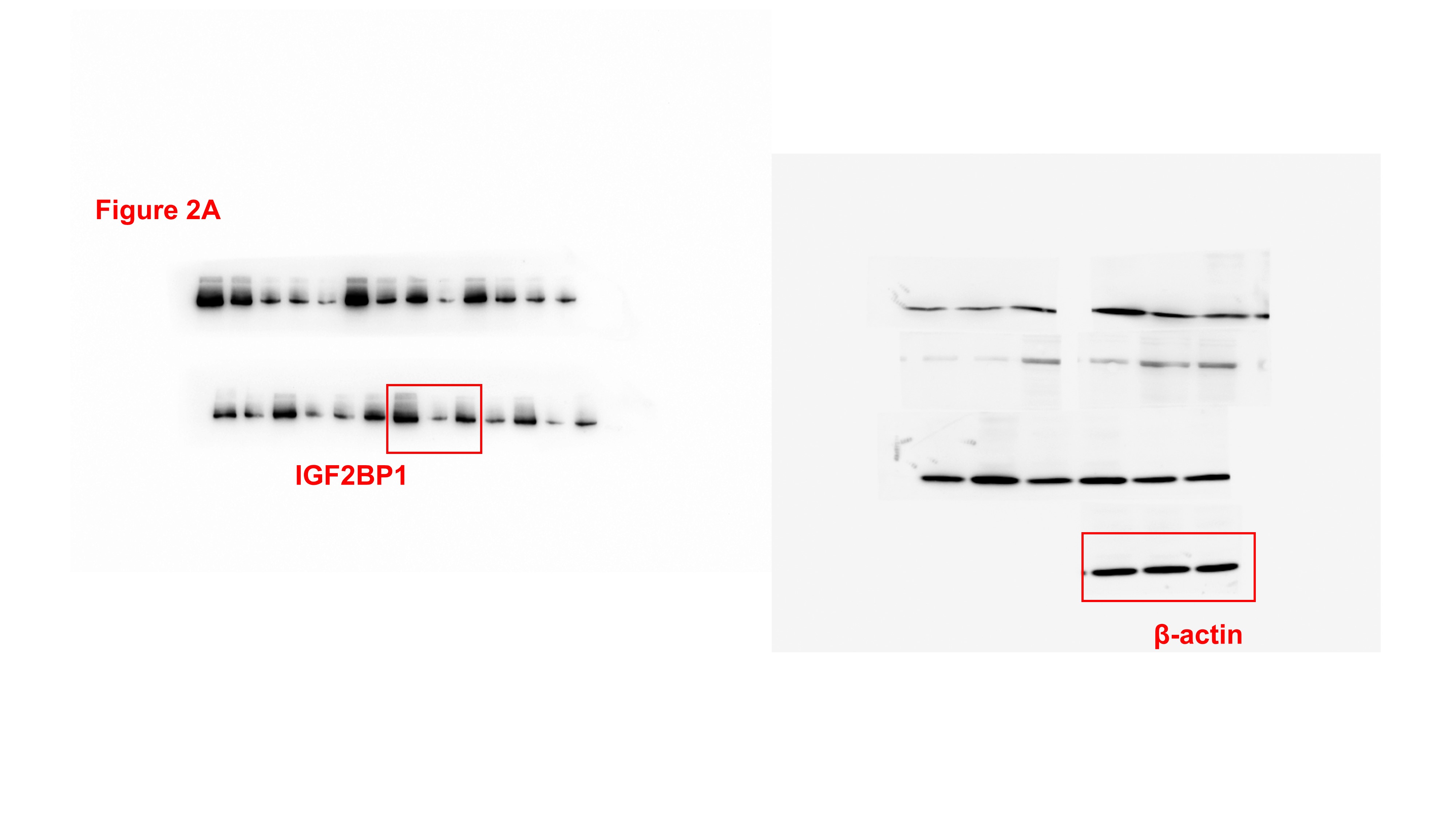

Supplement: Supplemental Information 2 [file peerj-11-14954-s002.zip › Label for WB blot/WB in Fig 2A .jpg]

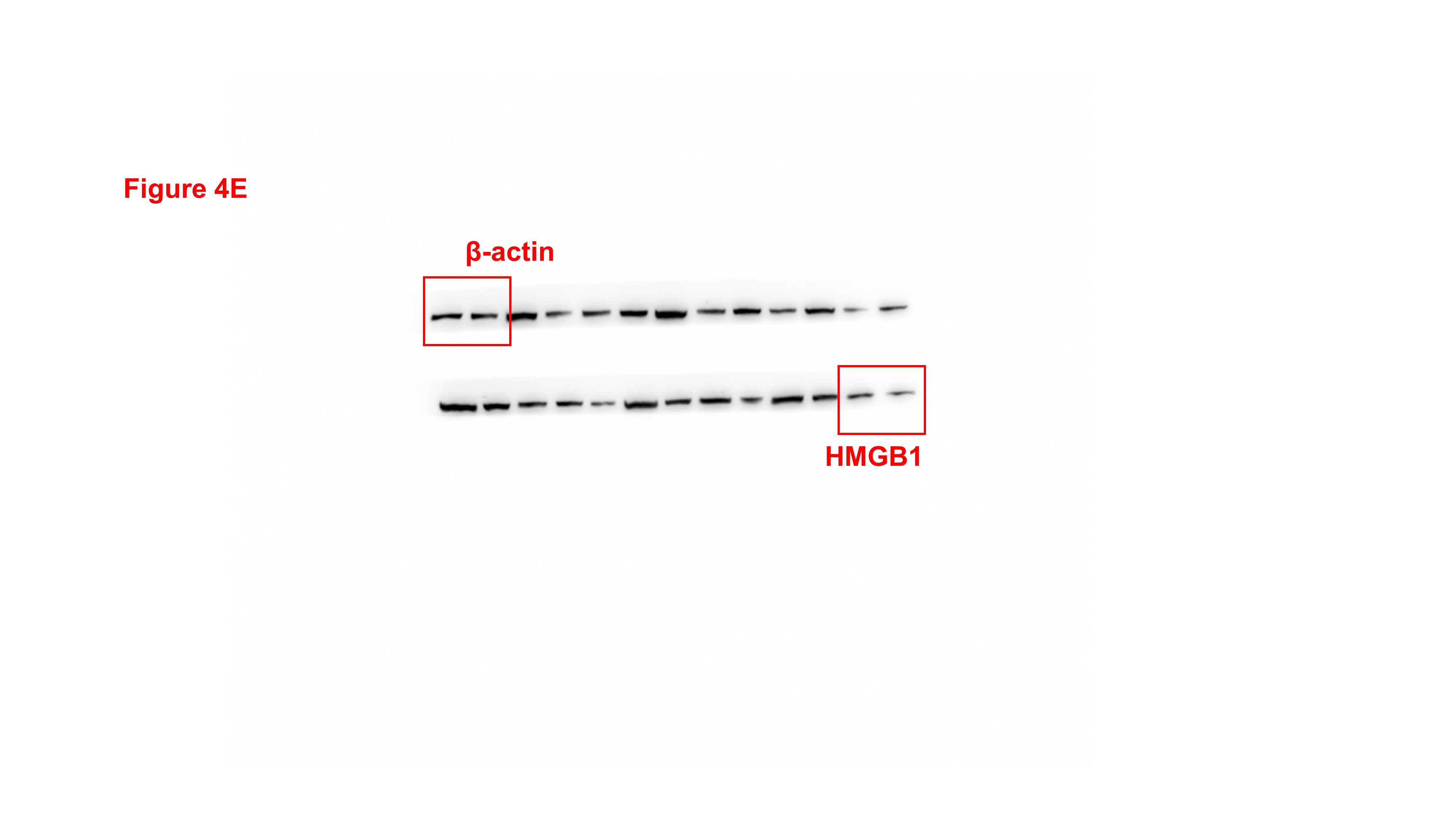

Supplement: Supplemental Information 2 [file peerj-11-14954-s002.zip › Label for WB blot/WB in Fig 4E .jpg]

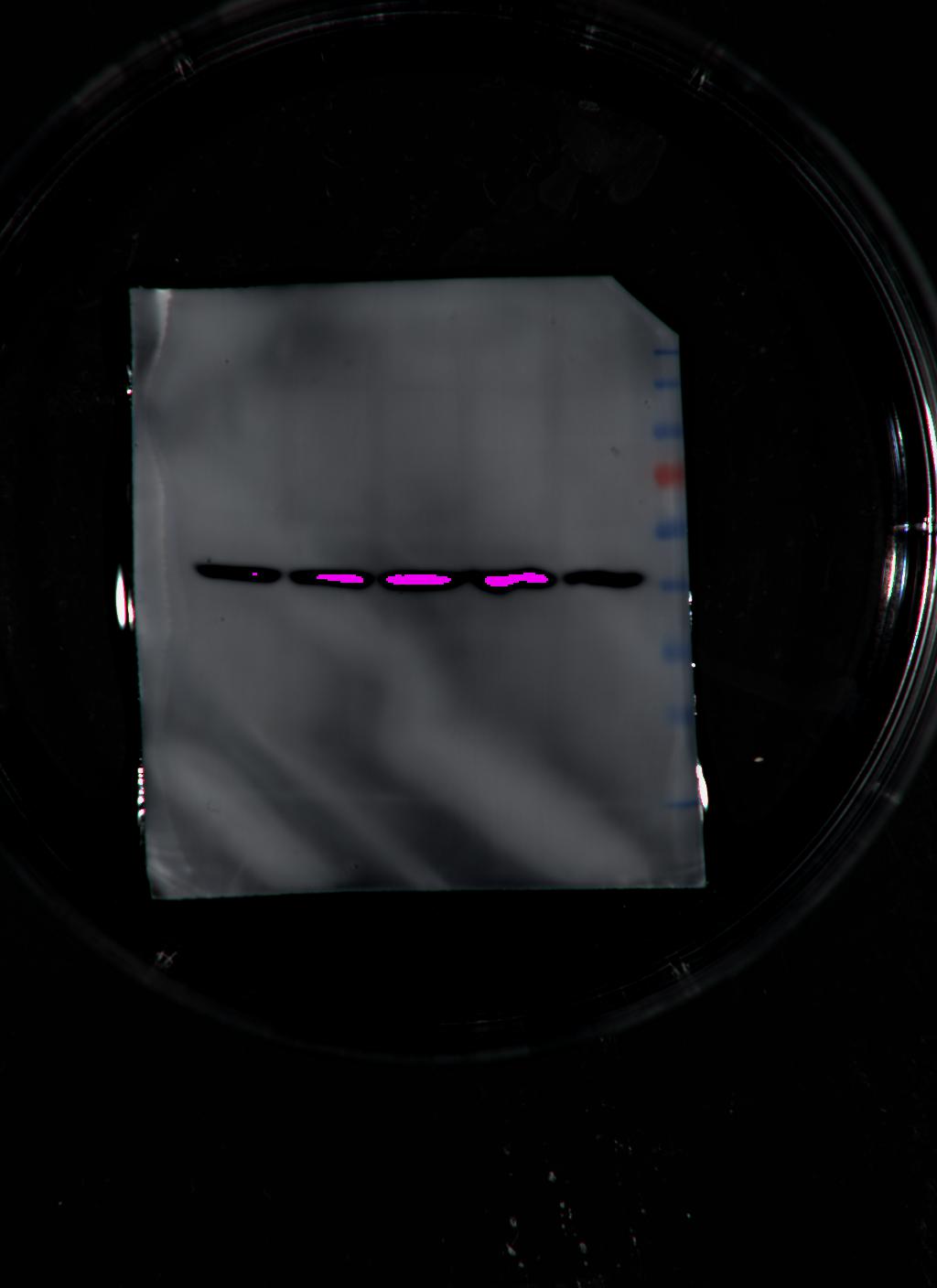

Supplement: Supplemental Information 3 [file peerj-11-14954-s003.zip › Uncropped Gels Blots/1-1-1 +Marker.jpg]

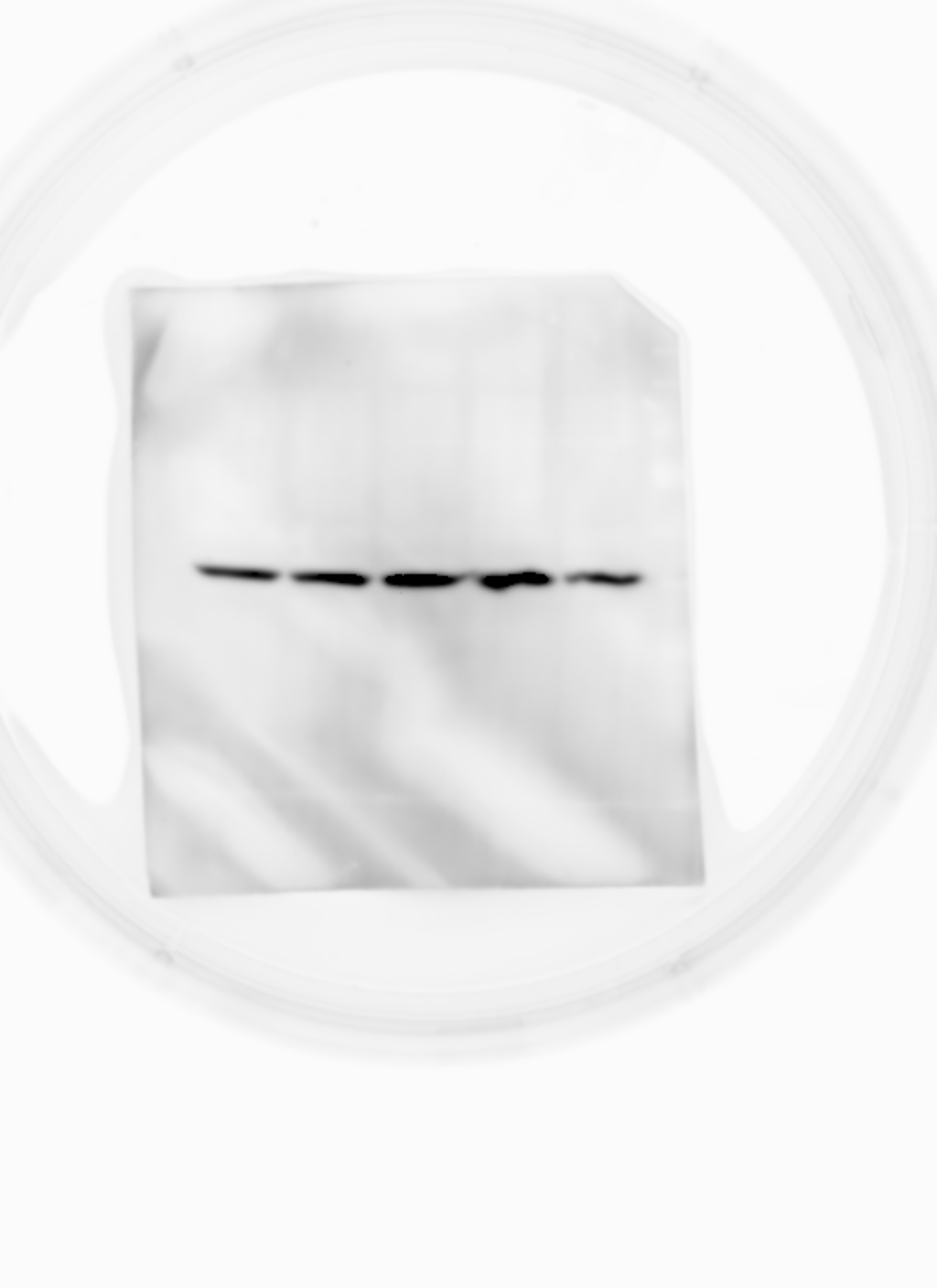

Supplement: Supplemental Information 3 [file peerj-11-14954-s003.zip › Uncropped Gels Blots/1-1-2 .tif]

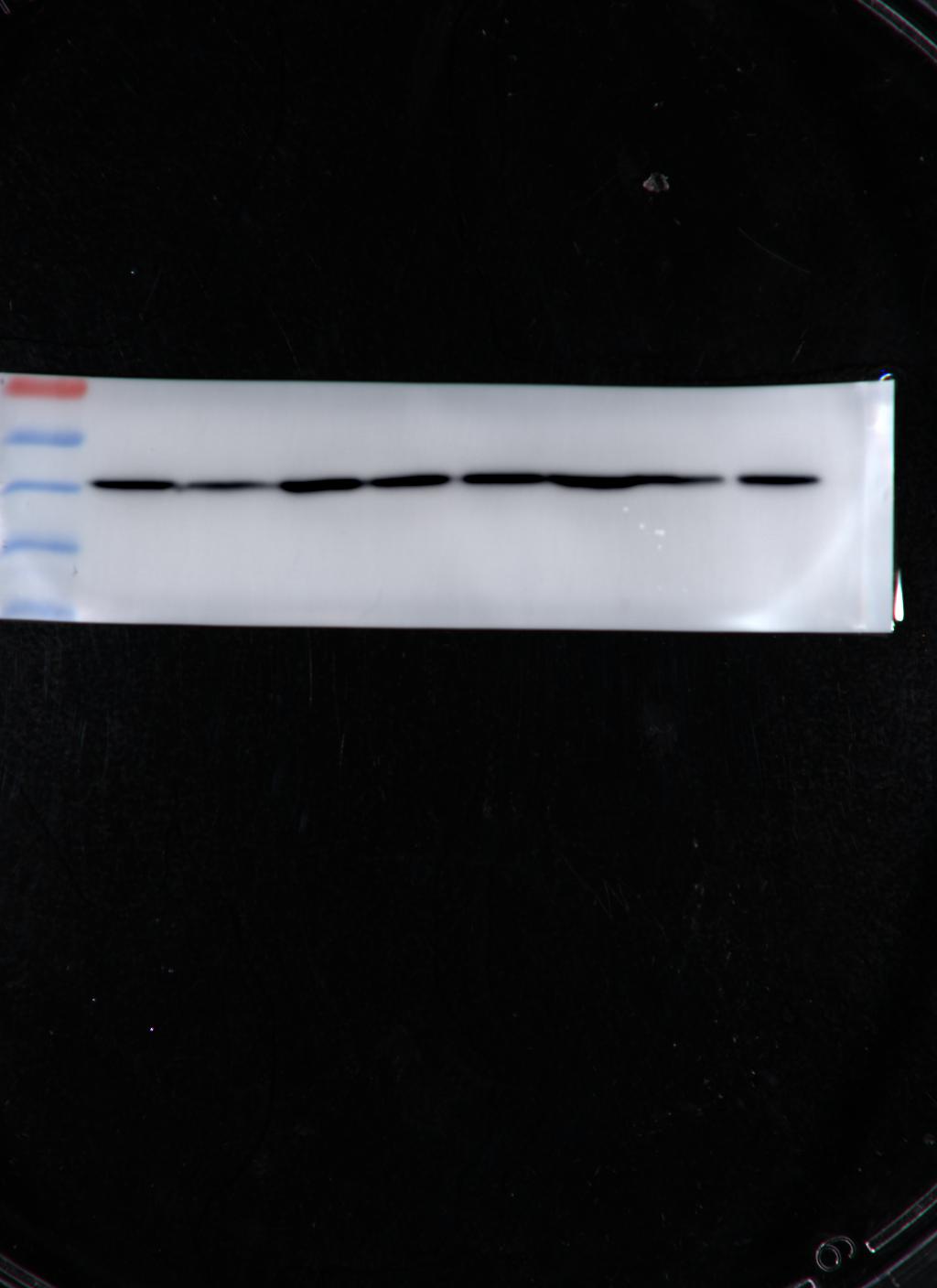

Supplement: Supplemental Information 3 [file peerj-11-14954-s003.zip › Uncropped Gels Blots/4-1 .jpg]

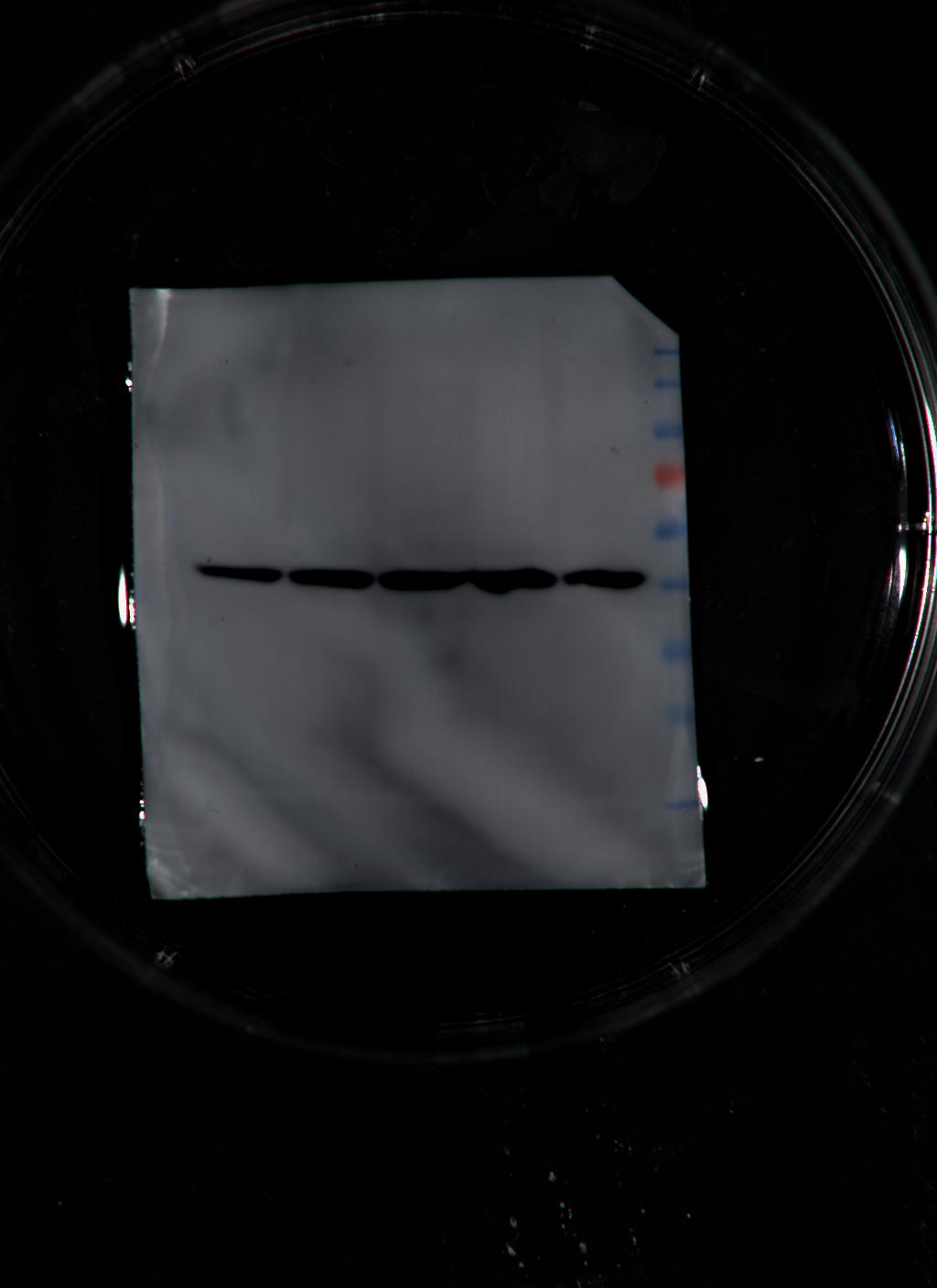

Supplement: Supplemental Information 3 [file peerj-11-14954-s003.zip › Uncropped Gels Blots/4-2 actin +Marker.jpg]
